# Genetic susceptibility to cervical neoplasia

**DOI:** 10.1016/j.pvr.2019.04.002

**Published:** 2019-04-05

**Authors:** Matthew A. Brown, Paul J. Leo

**Affiliations:** Institute of Health and Biomedical Innovation, Queensland University of Technology, Translational Research Institute, Princess Alexandra Hospital, Woolloongabba, QLD, 4102, Australia

**Keywords:** Cervical neoplasia, Heritability, Genomewide association study, HLA, Genetic risk prediction

## Abstract

Twin and family studies suggest that genetic factors play a role in cervical neoplasia susceptibility. Both rare high penetrant and common low penetrant host genetic variants have been shown to influence the risk of HPV persistence, and common variants have been shown to influence the risk of cervical neoplasia. The strongest associations with cervical neoplasia are with HLA genes, with associations having been demonstrated to both reduce and increase the risk of the disease. Fine-mapping using imputed amino-acid sequences of HLA-types has shown that the HLA associations are driven primarily by the HLA-B amino acid position 156 (B156), and HLA-DRB1 amino acid positions 13 and 71. This is informative about the types of peptides that may be useful for peptide vaccines. As cervical neoplasia is at least moderately heritable, genetics may be able to identify those at high or low disease risk. Using the findings of hundreds of disease-associated SNPs to calculate genetic risk scores, it has been shown that women with genetic risk scores in the bottom 10% of the population have very low risk of cervical neoplasia (<0.17%), whereas those in the top 5% have 22% risk of developing the disease. Further large scale genetic studies would be helpful to better define particularly the non-MHC component of genetic risk.

Whilst a high proportion of women are exposed to cervical human papillomavirus (HPV) infection, even in the pre-vaccine era only a small minority (∼1%) go on to develop cervical neoplasia [1]. Several environmental risk factors have been demonstrated to influence this risk, but genetic factors are also major determinants of those that are likely to proceed to cervical neoplasia. Identifying and understanding those determinants may assist in development of improved predictive or diagnostic tests, and of therapies or preventative approaches to the disease.

## 1 Heritability

Heritability in this setting refers to the proportion of the risk of developing a condition which is due to genetic factors. Traditionally this has been assessed using twin studies comparing concordance rates in monozygotic (MZ) and dizygotic (DZ) twin pairs, or in families. A systematic review of twin studies identified three studies of twin concordance in cervical cancer, with a pooled concordance rate of 0.12 for MZ and 0.08 for DZ twin pairs (P = 0.03) indicating moderate but statistically significant heritability of cervical cancer [2], marginally supporting a role for genetic factors in disease. Family studies have reported heritability to be 27–64% [3,4].

These study designs have potential weaknesses, such as ascertainment bias (typically an increased bias to study concordant twin pairs or multicase families), and assumptions made about environmental sharing within families that may not be correct. Further, their sample size is typically modest. Methods have recently been developed to assess overall genetic similarity between apparently unrelated individuals with and without disease in genome-wide association study data, from which the heritability captured by the SNPs genotyped can be assessed. This approach allows large sample sizes to be studied and is therefore more representative of the general population. When applied in cervical neoplasia, common variant (ie captured by the SNPs genotyped) heritability was shown to be 36% (SE = 2.4%) [5], consistent with the family studies, and slightly higher than a previous study using unimputed SNP microarray data with less genetic coverage (24% [6]).

## 2 Monogenic forms

A small fraction of patients with persistent HPV infection harbour mutations in single genes. However, the characteristic features of these monogenic disease forms, summarized in (Table 1), do not include cervical neoplasia for which no monogenic disorders have been reported either. Indeed none of the genes shown to be mutated in these diseases have been convincingly shown to be associated with cervical neoplasia in the general population.

**Table 1:** Known monogenic forms of chronic HPV persistence. EVER refers to ‘Epidermodysplasia verruciformis’. WHIM refers to ‘warts, hypogammaglobulinemia, infections and myelokathexis syndrome’ and ANKRD26 to Ankyrin’ Repeat Domain-Containing Protein 26’.

	EVER1	EVER2	EVER3	WHIM syndrome	ANKRD26 disease
Mutated gene	*TMC6*	*TMC8*	*CIB1*	*CXCR4*	*ANKRD26*
Associated features	Cutaneous warts, frequent malignant transformation	Cutaneous warts, frequent malignant transformation	Cutaneous warts, frequent malignant transformation	Cutaneous warts, hypogammaglobulinemia, immunodeficiency, myelokathexis syndrome Occasional associated developmental disorders e.g. tetralogy of Fallot.	Cutaneous warts
OMIM reference	226400	618231	618267	193670	610855 (gene only)

## 3 Common disease genetics

Monogenic diseases are caused by mutations with large effect on single genes, whereas most human disease is caused by an accumulation of common genetic variants each with individually small effect. The advent of GWAS has revolutionised the study of common diseases, providing robust assessment and control for population stratification, identification of cryptic relatedness, accurate genotyping through SNP microarray technology, and use of stringent statistical thresholds ensuring low false positive rates. A very large number of candidate gene association studies have been performed in cervical cancer [7]. Unfortunately this type of study has a high false positive rate due to failure to assess and control these factors, and therefore will not be considered further in this review.

At the time of submission of this article, five GWAS have been performed in cervical neoplasia (Table 2). The study by Leo et al. had adequate power to dissect the MHC associations [5]. Two protective HLA haplotypes were defined, one driven by association with *HLA-B15*, the second by a haplotype HLA-DRB1*13-DQB1*0603-DQA1*0103. The second haplotype has also been shown to be protective for oropharyngeal cancer [8]. Three haplotypes, HLA-DRB1*15/HLA-DQB1*0602/HLA-DQA1*0102, HLA-B*0702/HLA-C*0702, and HLA-DRB1*0401/HLA-DQA1*0301, were associated with increased risk of cervical neoplasia. Further analyses of association of the amino-acid makeup of the risk and protective haplotypes showed that the associations were driven by at HLA Class I HLA-B amino acid position 156 (B156), and at HLA-DRB1 amino acid positions 13 and 71. B156 is a tryptophan on the protective HLA-B*15, but either arginine, leucine, or aspartic acid in other HLA-B alleles. Whilst this amino acid is not in the peptide binding grove, it has been shown to influence antiviral immunity [9], presumably through conformational effects on the HLA-B protein affecting peptide presentation properties. HLA-DRB1 positions 13 and 71 lie in pocket 4 of the peptide binding grove, and therefore likely to influence peptide presentation. These findings are of particular importance in research of potential peptide based therapies or vaccines for cervical neoplasia, as they could assist in selection of optimum peptides, and also suggest that it is likely that peptides which are handled differently by these alleles will have different therapeutic benefits or vaccine efficiency.

**Table 2:** Genomewide association studies to date in cervical neoplasia. Nil significant indicates no significant genomewide significant findings.

	Chen et al., – 2013 [10]	Chen et al., – 2016 [11]	Miura et al., – 2014 [12]	Shi et al., – 2013 [13]	Leo et al., – 2018 [5]
Sample size, discovery phase	1075 cases, 4104 controls	1634 cases, 4442 controls	226 cases, 186 controls	1364 cases, 3028 controls	2866 cases, 6481 controls
MHC findings	Strong MHC association, implicating MICA5.1 and rs9272143 (eQTL for HLA-DRB1)	Replicates 2013 study.	Nil significant	Strong MHC association, peak between HLA-DPB1 and –DPB2.	Strong MHC association as discussed below.
Non-MHC findings	Nil significant	Nil significant	Nil significant	4q12 near EXOC1, 17q12 near GSDML	Nil significant, including specifically not at EXOC1 or GSDML

To date no convincing non-MHC genetic association that has been replicated between studies has been reported, with the possible exception of rs8067378 at 17q12, where inconsistent findings have been reported. Leo et al. demonstrated that the vast majority of the common variant heritability of cervical neoplasia was non-MHC encoded. The failure to identify variants is likely a question of power. As all studies have used general population controls a significant proportion of whom likely have persistent HPV infection, the study power is likely to have been only moderate for each individual study. A meta-analysis of these studies therefore may well be more informative than the individual studies have been.

## 4 Genetic risk prediction

Although individual genetic markers associated with cervical neoplasia do not provide enough information to stratify women according to risk of cervical neoplasia, Leo et al. demonstrated that using multiple markers, that high and low risk groups could be identified. Women with genetic risk scores in the bottom 10% of the population were shown to have a risk of cervical neoplasia of <0.17%, compared with an estimated 1% of women with HPV who develop neoplasia. In contrast, women in the highest 10% of genetic risk scores were shown to have approximately >7.5% risk of developing cervical neoplasia, and in the top 5% have 22% risk of developing the disease. Given that nearly 20 million Americans have had SNP microarray genetic testing performed by direct to consumer testing companies and have the data required for this test already available, implementation of this test could identify many women who are at quite high disease risk at low cost. Further validation of these findings, including in general population cohorts, is warranted.

## COI statement

Neither MAB nor PL have any conflict of interest in regards this publication.
